# Sustainable Materials and their Contribution to the Sustainable Development Goals (SDGs): A Critical Review Based on an Italian Example

**DOI:** 10.3390/molecules26051407

**Published:** 2021-03-05

**Authors:** Elza Bontempi, Giampiero P. Sorrentino, Alessandra Zanoletti, Ivano Alessandri, Laura E. Depero, Andrea Caneschi

**Affiliations:** 1 INSTM and Chemistry for Technologies Laboratory, Department of Mechanical and Industrial Engineering, University of Brescia, via Branze, 38, 25123 Brescia, Italy; g.sorrentino002@unibs.it (G.P.S.); alessandra.zanoletti@unibs.it (A.Z.); laura.depero@unibs.it (L.E.D.); 2 INSTM and Chemistry for Technologies Laboratory, Department of Information Engineering, University of Brescia, via Branze 38, 25123 Brescia, Italy; ivano.alessandri@unibs.it; 3 Dipartimento di Ingegneria Industriale-Dief, Università Degli Studi di Firenze, INSTM Research Unit of Firenze, Via di Santa Marta n. 3, Firenze 50139, Italy; andrea.caneschi@unifi.it

**Keywords:** sustainable materials, SDGs, circular economy, raw materials

## Abstract

The Sustainable Development Goals (SDGs) have been proposed to give a possible future to humankind. Due to the multidimensional characteristic of sustainability, SDGs need research activities with a multidisciplinary approach. This work aims to provide a critical review of the results concerning sustainable materials obtained by Italian researchers affiliated to the National Interuniversity Consortium of Materials Science and Technology (INSTM) and their contribution to reaching specific indicators of the 17 SDGs. Data were exposed by using the Web of Science (WoS) database. In the investigated period (from 2016 to 2020), 333 works about sustainable materials are found and grouped in one of the following categories: chemicals (33%), composites (11%), novel materials for pollutants sequestration (8%), bio-based and food-based materials (10%), materials for green building (8%), and materials for energy (29%). This review contributes to increasing the awareness of several of the issues concerning sustainable materials but also to encouraging the researchers to focus on SDGs’ interconnections. Indeed, the mapping of the achievements can be relevant to the decision-makers to identify the opportunities that materials can offer to achieve the final goals. In this frame, a “Sustainable Materials Partnership for SDGs” is envisaged for more suitable resource management in the future.

## 1. Introduction

The 17 Sustainable Development Goals (SDGs) with their targets are at the heart of the United Nations 2030 Agenda for Sustainable Development [1] and were adopted by the 193 member states of the United Nations General Assembly, in 2015. SDGs were proposed to introduce fundaments in sustainable development, with the aim to progress towards health and well-being worldwide, promoting the ending of all forms of inequalities and poverty, supporting climate actions, quality education, gender equality, peace, and social justice. The goals are explicitly stated in their targets (ranging from five to twelve targets per goal).

SDGs are derived from the three pillars of sustainable development: the economic, social, and environmental pilasters, to achieve the sustainability of humankind on Earth.

The SDGs are generally presented and discussed as separate goals, but several of them are interrelated, with complex and not always evident correlations that may generate positive [2] or negative connections [3].

In particular, the SDGs’ objectives are strictly connected with all the research disciplines (humans, engineering, medical, statistics, economic, and so on), and must be the focus for the science and technology of the future. Indeed, the complexity and multiple dimensions of the SDGs’ targets very often need the contributions of different disciplines and a multi-trans-disciplinary approach. 

However, although SDGs are recognized to be a priority, their implementation is not progressing as expected and advances at different rates and with several difficulties, also due to the current pandemic context. Indeed, COVID-19 has produced an unexpected crisis at the global level, with a tragic impact on the life and health of almost all the world population [4]. The pandemic has slowed economic growth, increased unemployment, and raised poverty and starvation [5]. The resulting global recession is alarming and has opened some doubts about the possibility and the ways to purse the SDGs in the post-pandemic age [6]. In particular, just two of the 17 SGDs (eliminating preventable deaths among newborns and under-fives and getting children into primary schools) appear to be achievable in time. 

However, the pandemic has also demonstrated that SDGs go beyond national strategies. Then, the SDGs’ universal nature and their interactions need to be properly considered globally by all policymakers; thus, it is necessary to propose a suitable framework to identify the possible interactions among all the SDGs and their indicators that may be proposed internationally [7]. 

There are several papers, reviews, and discussion works devoted to SDGs (see for example [8,9]). Moreover, the possibilities offered by specific research to archive globally all SDGs are poorly investigated. The framework proposed in this work may be an example of a suitable strategy to promote and encourage possible synergistic activities that may result in the SDGs’ acceleration and progress. 

This work is expressly devoted to materials, because they play a fundamental role in all the aspects of humans life: their consumption is rapidly rising in the last years, reaching an unsustainable material footprint per capita, which is still expected to increase in the next decades [10]. In particular, materials’ extraction, manufacturing, consumption, and end-of-life options strongly impact the economic, social, and environmental pillars [11]. 

**Figure 1:**
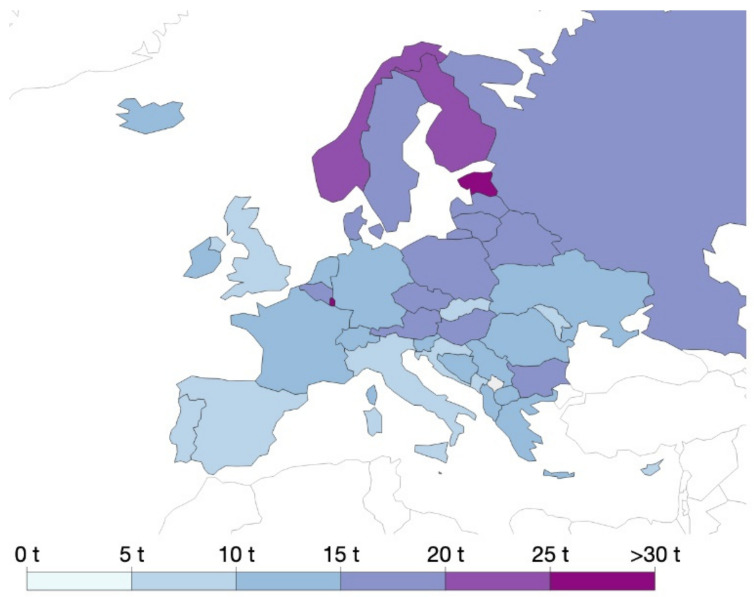
Domestic material consumption per capita, measured in tons per person per year (data refer to 2017). Data from “Our World in Data“ [12].

Figure 1 summarizes domestic material consumption in Europe in 2017. A very recent United Nations report (of 28 April 2020) highlights that domestic material consumption per capita in Europe is about 40% higher than the global world average, strongly suggesting the necessity to better manage resources and reduce consumption in the future.

Clearly, materials are strictly connected to several of the SDGs. For example, raw materials extraction, which should be linked to the well-being of the population living in a territory rich in natural resources, can generate pollution, GHG emissions, and stress on Earth. Advanced materials also play a fundamental role in technology developments, innovation, and resources in all fields (from medicine to engineering and agriculture to artificial intelligence).

This suggests a strict interlinkage among the different SDGs and the various steps of raw materials management. Then, the substitution of raw materials with sustainable materials is mandatory for global sustainability [11]. 

Currently, the link between materials and SDGs’ targets has not been studied in detail, while this topic needs more attention and insight. 

In this review, the interconnection between materials and SDGs, their potential contributions, and their impacts is discussed, analyzing the research activity made in the frame of the Italian Interuniversity Consortium of Materials Science and Technology (INSTM). 

In accord with the Web of Science (WoS) [13] database, Italy is one of the most productive countries in the European Union on the topic of “sustainable materials”, together with Germany and Spain.

In particular, INSTM, which involves researchers from 50 Italian universities, is very active, and it can be considered a representative group concerning the Italian research on this topic.

Based on the INSTM activities, we will show the interconnections between materials research and SDGs. The INSTM publications can be considered an example, showing the role in SGDs achievement and demonstrating that specific goals can be achieved only if strategies about materials are identified, clarified, and supported.

The aim of this review paper is to use selected papers about materials and sustainability, not only to highlight the materials’ importance to promoting sustainable development, but also to offer an unconventional and applicative vision about SDGs’ interconnection. In particular, even if some SDGs seem to not involve materials, an indirect link can be always established. However, thanks to the materials’ example, this paper also shows that sustainability is based on pillars that can involve different principles in comparison to the circular economy. This, for example, can better allow understanding of why to promote suitable measures in a sector (to support a specific goal); it is mandatory to foresee also interventions in other sectors.

### Materials and Sustainable Development Goals (SDGs)

“Raw materials” are fundamental for several industrial sectors, like chemicals, forestry and agriculture, medicine, industrial metals, and mining. Natural resources are mined and manufactured, with generally high impacts on the natural environment. Often raw materials are obtained in areas of conflict or jurisdictions where there is poor enforcement of labor, environmental, or business integrity standards [14]. There are over 100,000 materials in our world [15], and suitable materials selection is critical. Different factors can be considered to select among alternative materials, depending on the required functional properties and the final cost. Today, in the frame of SDGs, more attention must be devoted to sustainability. Sustainable materials can be defined as materials derived from renewable resources. They must have a zero/minimal impact on the environment and society for their extraction and production [16]. Examples are recycled metals, bio-based polymers, and materials for renewable energy. Generally, people are more and more aware of the environmental dimension of the sustainability issues and, even if the economic dimension of sustainable development is generally also underestimated, the social dimension is the less known pillar. “Social sustainability” is linked to the social outcomes and values, such as equality, social responsibility, children′s work, gender equality, community resilience, freedom from poverty, and so on [17]. These are the harder to define and quantify sustainability issues, which can be summarized by the “inclusive growth” concept, i.e., economic growth must be fairly shared across society members and create opportunities for all. In this context, it is important also to highlight that it is not possible to completely avoid mining. Raw materials have been essential for humans to live on this planet and have been since the dawn of humanity. Moreover, it is now evident that the extraction of some quantities of particular minerals containing specific elements, such as copper and rare earth elements, will be also mandatory to deploy renewable energy technologies, for substantial progress against greenhouse gas reduction goals [18]. Mining raw materials is still fundamental; then, in some cases, a relevant question should be not only related to whether such materials can be provided, but rather at what cost—inclusive of environmental and social factors—they can be provided [18]. Moreover, metals recycling can improve the metals’ future availability by decreasing the request for virgin resources. The United Nations reports: “Sustainable consumption and production are about promoting resource and energy efficiency, sustainable infrastructure, and providing access to basic services, green and decent jobs and a better quality of life for all. Its implementation helps to achieve overall development plans, reduce future economic, environmental and social costs, strengthen economic competitiveness, and reduce poverty” [19]. Then, sustainable materials and innovation in their productions must be in accord with SDGs, and this review shows they are connected (directly or indirectly) with all the goals.

## 2. Methodology

The WoS database [20] was used to map the publications of the INSTM consortium. The selected temporal range was from 2016 to 2020 to account for the adoption of the SDGs in 2015. The selection of the most recent research ensures that the selected papers aligned with the principles of sustainable development. This papers’ selection was performed by choosing the topic “sustainable or sustainability” in “all fields” in the database. In particular, the following affiliations “Consorzio Interuniversitario Nazionale per la Scienza e Tecnologia dei Materiali or INSTM” were considered. The search resulted in 333 peer-reviewed papers (113 are open access). The list of all these articles is reported in the supporting information (Table S1). All these papers were considered and analyzed in detail to obtain a list of all associated SDGs.

**Figure 2:**
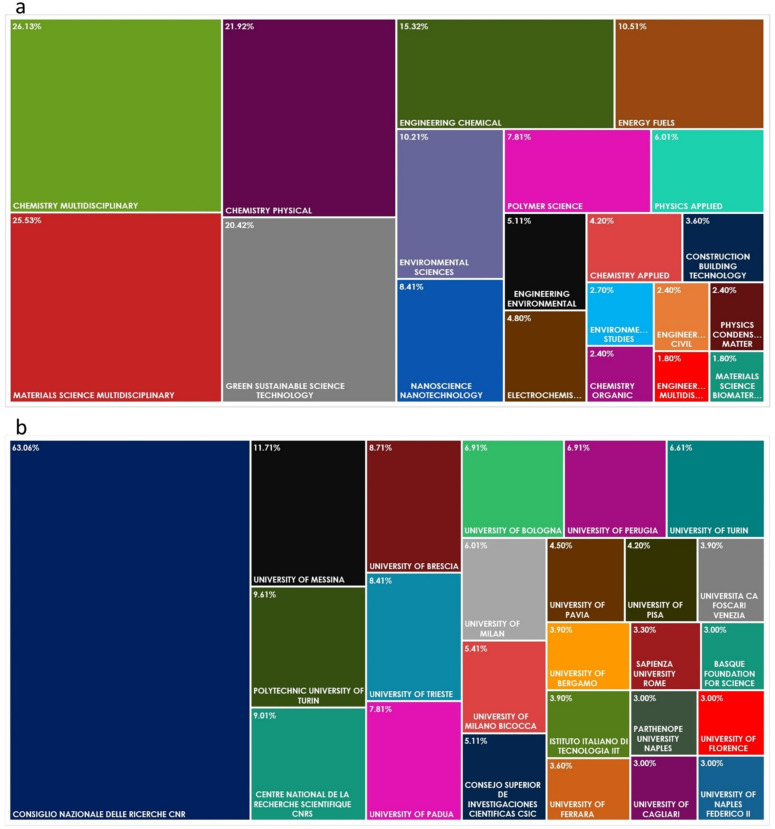
Web of Science (WoS) categories of sustainable materials (**a**) and the INSTM research units working on sustainable materials topic (**b**). Publications from 2016 to 2020 were considered.

Figure 2a shows the resulting WoS categories of sustainable materials investigated in the above described 333 papers. In this figure, only the first 20 categories defined by WoS are reported. Figure 2b shows the INSTM Research Units and collaborating research organizations involved in the research developed in the 333 selected papers. In this figure, only the first 25 organizations identified by WoS are reported. Figure 2 helps in understanding the pivot role for clustering this research performed by INSTM, through the networking of its Research Units and by means of the establishment of collaborations with important Italian and foreign research institutions.

All the works were grouped into the following six main categories: (1) chemicals, (2) composites, (3) novel materials for pollutants sequestration, (4) bio-based and food-based materials, (5) materials for green building, and (6) materials for energy. 

The paper is organized in the following way: Section 1.1 briefly introduces the link between materials and sustainability; Section 2 defines the methods used to select the literature papers; Section 3 reports the INSTM literature analysis results, with the paragraphs devoted to the specific selected sustainable materials categories (Section 3.1 Chemicals, Section 3.2 Composites, Section 3.3 Novel Materials for Pollutants Sequestration, Section 3.4 Bio-Based and Food-Based Materials, Section 3.5 Materials for Green Building, Section 3.6 Materials for Energy); Section 4 better highlights and specifies the interconnection between SDGs and sustainable materials; and finally in Section 5 and Section 6, the future perspectives and conclusions of this work are given.

## 3. Sustainable Materials Developed by the INSTM Consortium

The world economy is still dominated by a linear path where extraction–consumption–disposal makes several processes unsustainable. SDGs, in accordance with the circular economy principles, aim to save resources and maintain the products for as long as possible, in an active stock, then recovering and regenerating materials at the end of their life (see for example SDGs 8–9–12). These goals are strictly connected with sustainable materials development. To highlight the influence on the SDGs, the papers (identified by DOI name) regarding sustainable materials developed by INSTM researchers have been grouped into six categories: chemicals (33%), composites (11%), novel materials for pollutants sequestration (8%), bio-based and food-based materials (10%), materials for green building (8%), and materials for energy (29%) (see Table 1).

### 3.1. Chemicals

The goal of zero hunger (SDG 2) strongly depends on agricultural productivity. During the next decades, sustainable fertilizers will globally have great potentialities in enhancing agriculture and food security, providing not only materials able to restore soil characteristics but also free of contaminants, such as heavy metals [21]. Sustainable materials, such as phosphates [22], were already developed for agriculture to achieve innovative nanopesticides and nanofertilizers, with limited phytotoxicity and human side effects [23,24]. Researchers designed a formulation able to significantly influence sorption and degradation phenomena with low environmental impact. Moreover, new sustainable chemicals are expected to play significant roles in all the economic fields. Indeed, according to the introduction of stringent legislations recently promoted by several countries, some chemicals, such as halogenated compounds (for example brominated diphenyl derivatives), have been banned, due to their demonstrated high toxicity for both humans and animals [25]. The persistence in the environment of these compounds stimulated the researchers to develop safe substitutional materials [26] (SDGs 14–15). In this frame, for example, the production of phosphorus compounds as flame retardants allowed the reduction of halogen-based additives [27]. Some chemicals, for example, hydrolysable tannins [28], can be obtained by extraction from biomass, by sustainable processes, making available valuable products to be used for example as radical scavenging, antimicrobial agents, and antioxidant, at low environmental costs. Acetonitrile, which is one of the main solvents used for pharmaceutical and laboratory applications, was obtained by ethanol and ammonia [29] (SDGs 1 and 2), instead of propylene. Based on LCA evaluation, this represents a sustainable new process, with the possibility to obtain a complete materials recovery, exploiting renewable sources.

### 3.2. Composites

In Europe, packaging represents 39.6% of the total plastics (that often are composites materials) demand, highlighting that packaging is the most important sector for the plastics request [30]. It is evident that for Europe, packaging has the drawback of plastic wastes disposal. As a consequence, the packaging sector has an urgent need to implement new sustainable packaging materials [26], derived from renewable sources. The research activity concerning alternative packaging (involving SDGs 2–9–11–12–14–15) obtainable from sustainable resources (for example starch, poly(lactic acid), chitosan), is currently very active, with the main aim to find suitable alternatives to polymers derived from fossil fuels [31]. Cellulose is another material that can be used to realize new sustainable composites [32,33], able to produce films with several applications, where the biodegradability is a request [34], such as single-use goods (SDGs 1-2-7). Another urgent need is to create also new fillers (and not only matrices) to replace natural ones, such as calcite [26]. For example, nanocellulose crystals can be obtained [35] as fillers able to enhance several properties such as mechanical, barrier, and thermal properties, surface wettability, and drug release characteristics. Other sustainable composites have been obtained by agro-food waste (for example coffee silverskin, which is a waste derived from coffee), and used as reinforcing agents in biopolymer-based materials [36]. Finally, stabilized fly ashes were employed as a calcite (or talk) substitute to realize polypropylene composites [37], showing that the mechanical properties of final products can be maintained.

### 3.3. Novel Materials for Pollutants Sequestration 

Natural resources mining was always associated with terrestrial ecosystem degradation. In the frame of SDGs 14 and 15, restoring degraded landscapes and preserving water systems are urgent priorities. Several sustainable materials to depollute air and water have been developed by INSTM researchers. For example, particulate matter (PM) is one of the main contributors to air pollution. It is composed of a complex mixture of organic and inorganic solids and liquid particles suspended in the air, very often originated by anthropogenic activities. Its persistence can be exacerbated by the particular geophysical and microclimatic characteristics of the polluted area [4]. The European Environment Agency reported that in 2013, about 467,000 premature deaths in Europe were attributed to PM_2.5_ pollution [38], often due to anthropogenic activities. Recently, starting from alginate and waste silica sources, some porous materials have been developed [39], able to sequestrate fine airborne particles [40]. These materials, which can be used for example as plaster, can contribute to SDG 11, allowing the achievement of sustainable and resilient cities [41]. Moreover, also the global warming issue is related to the anthropogenic emissions that are discharged into the air. In particular, greenhouse gases refer to several gases that have direct effects on climate change. The emission of greenhouse gas increased worldwide in the last decades. This was mainly attributed to fossil fuel combustion. Indeed, carbon dioxide is the most abundant and known gas linked to the increase in world temperature. The mineral carbonation can be considered a sustainable technology able to use carbon dioxide and waste as reagents. It was shown that it can be a permanent solution for CO_2_ sequestration, comparable to geological and ocean storage [42]. This technology, if based on waste recovery, requires low-energy paths (SDGs 7-11-13). CO_2_ can also be a tool for developing an industrial symbiosis strategy: strategic partnership among industries can be found by the industrial waste reuse in the production of new materials for different industrial sectors (CO_2_ can be considered a waste for some industrial activities, but also a raw material for some companies, using this by-product, for example, for chemicals synthesis). The development of new catalysts and smart interfaces can drive CO_2_ towards the selective formation of C1-C3 chemicals [43]. In this frame, the research on sustainable materials can provide novel strategic routes and technologies [44] (SDG 9). Concerning water, many communities around the world rely on scarce and polluted water sources, often far from the homes and, normally, with the water supply assigned to women, leaving them vulnerable and exposed to different dangers (SDG 5). One origin of water contamination is the lack of sanitation [18]. Sustainable materials can offer several opportunities to depollute water, as the synthesis of safe adsorbent materials derived from waste [45,46]. New strategies for pollutants removal allowed also the synthesis of alginate-derived blend [47], obtained without any commercial additives. These new materials show a high performance in the sequestration of pollutants in water matrices (SDG 6). Recent works of INSTM research groups demonstrated that a rational design of remediation systems inspired by criteria of sustainability and green chemistry can help to advance detection and removal of a variety of persistent organic pollutants from water. In this context, alginate-based smart sponges play a key role and open exciting opportunities for future applications [47]. Additionally, porous materials were synthesized to obtain membranes and foams [48], with good results as an adsorbent to remove heavy metals. These materials are flexible and can be easily shaped or foamed into precast molds. Good mechanical properties of these materials are obtained by using metakaolin and polysiloxane oligomers, making the monolithic artifacts resistant to washings as well as to water adsorption and desorption. Other heavy metals stabilization technologies, based on the use of waste and by-products, were also proposed [49] to manage, for example, fly ash [44,50]. Finally, it is also interesting to highlight the new emerging water pollutants, typical of the richest countries, like for example pharmaceuticals [47,51]. Some of these pollutants are diffused in surface water and groundwater, due to their stability and hydrophilicity, making them able to persist also in wastewater treatment plants. This needs the development of devoted depolluting materials, able to remove them [51]. Significant step-forwards in the removal of persistent organic pollutants and, in particular, pharmaceutical compounds, have been recently achieved by INSTM research groups.

### 3.4. Bio-Based and Food-Based Materials

Valuable and most suitable ways to produce chemicals need the use of different synthetic routes, overcoming the disadvantages due to the use of commercial solvents and harsh conditions. Several examples are diffused in the biomaterials field, where materials are mainly synthesized to produce new sustainable and safe compounds for medical applications. For example, new sustainable strategies to convert polyesters into functionalized oligomeric derivatives that can be used for printing customized biomedical devices were recently proposed [52].

Following the principles of green chemistry, new drug delivery composites for the controlled release of antibiotics have been realized [53] (SDG 3), and new efficient drug delivery systems have been proposed. Other materials for diagnostic and therapeutic agents have been developed [54,55], with reduced toxicity and avoiding some undesired side-effects of the chemicals used in medicine. In some cases, water has been used as a green solvent instead of less sustainable commercial reactants (SDG 12).

Some of these materials can be obtained from wastes, by-products, or crops, hence contributing to reaching also the objectives of SDG 11.

The chitosan and its derivatives [56] have been used in several biomedical and pharmaceutical applications, and also as textile and in wastewater treatments. Between one third and one half of the world food production is not consumed [57,58]. In 2012, up to 30% of food production in the European Union was not consumed and, currently, 88 million tons of food is being wasted every year [57]. For those reasons, preventing and reducing food waste and finding alternative uses for it, are critical, urgent tasks, which should be faced from a circular economy viewpoint that considers food-discards as feedstocks for new materials.

In some cases, biomaterials can be interesting for combined applications, such as bio-remediation and fuel production. For this aim, a new chemistry approach, the so-called “Azure Chemistry”, was recently proposed [38], to restore or reconstruct the ecosystems by employing materials and technologies that are truly environmentally sustainable. For example, microalgal was used as filler in the production of bioplastic [59] (SDGs 14–15). To close the circle after their uses in bio-remediation, these microalgae can be used as a biofuel source [60] (SDG 11).

### 3.5. Materials for Green Building

One of the biggest impacts on the environment arises from the construction sector that contributes to 30% of raw material extraction. Moreover, this kind of industry is also responsible for 25% of solid waste generation, 25% of water use, and 12% of land exploitation [61] (SDG 15).

The demand for cement is constantly increasing, due to the growth of the world population, making it the most used building material, reaching a production of 10 billion tons per year. Because of these huge quantities, the impact on the environment is substantial in terms of embodied energy consumption, raw materials required, and greenhouse gas emissions. Indeed, the latter aspect amounts to around 5–7% of anthropogenic carbon dioxide emitted contributing to global warming, mostly because of the Portland cement, one of the widely used binders of modern concrete mixtures, which is not environmentally friendly [62] (SDG 13).

The building construction sector has the main challenge of satisfying at the same time both the need to reduce its impact on the environment and the growing demand for housing, as it is expected to reach 10 billion people worldwide by 2050. These targets should be reached through the approach to design based on the “3R—Green Strategy”: reduction in consumption of gross energy for construction materials production, reduction in polluting emissions, and reduction in consuming not renewable natural resources [63].

The achievement of Green Building must see the use of more efficient materials or better still of ones recovered from waste that would generally end up in landfills. It has been shown that the potential of mortars obtained by aggregates based on coarse recycled brick/concrete instead of natural ones can be a solution to avoid the depletion of natural resources and the disposal of waste material (SDG 15) [64]. Some researchers have investigated recycling automotive shredder residues and using the non-metallic fraction mixed with concrete, a lightweight aggregate with good mechanical properties is obtained [65]. 

Another material that is increasing in Europe is glass reinforced plastic (GRP), and research has shown that the performances of fired clay bricks can be improved by replacing the clay volume at 10% with glass reinforced plastic dust (GRPd). The final product has the feature to absorb heat and decrease brittleness, which is the main cause of breakage during transport [61].

Innovative systems with higher performances and lower environmental impacts for thermal and acoustic insulation of buildings have been studied to increase indoor comfort and decrease the energy used for heating the environment [66]. New geopolymeric foams, with interesting thermal and mechanical properties, have been obtained using coal fly ash (another waste material) and alkali silicate solution [67]. 

The quality of the indoor is closely linked to health, looking at both safety and comfort. Research has provided air purification by volatile organic compound and insulation using multifunctional materials made up of silica and titania [68] (SDG 3).

The external cladding of the buildings has the role to protect the structure from weathering and deterioration of the concrete, and therefore from the consequence of the corrosion of the internal reinforcement. Considerable studies have been conducted on a hybrid porous material based on waste material such as silica fume (a by-product of the silicon and ferrosilicon production) or bottom ash (derived by municipal solid waste incineration) used as a coating on the building’s facades and capable of adsorbing particulate matter [41] (see also Section 3.3). The research has demonstrated that new eco-coating types can give the buildings active participation in safeguarding the environment. This is the “Smart Cities” concept, where suitable urban development must be strictly related to suitable measures to reduce people′s exposure to pollutants [39] (SDGs 11–15).

### 3.6. Materials for Energy 

Renewable energies are expected to account for a growing economy in the next future, with the ambition to reach a net cut in CO_2_ emissions. Moreover, energy infrastructure will also need to be replaced, due to the necessity of new infrastructure for future energy sources.

In particular, energy transition towards a zero-carbon economy is mandatory to mitigate climate change by carbon dioxide emissions reduction [69]. Then, several resources of the current worldwide research are addressed to find alternatives for energy production and to identify suitable fuels able to avoid fossil sources drawbacks (SDGs 7–11). Some potential solutions are based on the production of new electrochemical devices from biowaste [60,70]. 

At the present, great efforts are devoted to the development of photo-electro-catalytic cells for the CO_2_ conversion, to produce solar fuels (such as methanol or longer carbon-chain products) [71] and more generally for the development of new technologies for the use of captured carbon dioxide [72,73], as already reported in Section 3.3 (SDG 13).

Hydrogen [74] is considered an unlimited raw material (because of the large abundance of water) having a specific energy density (between 120 and 142 MJ/kg) that is 2.75 times higher than that of other hydrocarbons [75]. Then, some researchers propose new strategies to obtain its production by using sustainable ways [76,77], such as starting from ethanol or water electrolysis driven by renewable sources [78]. However, these processes for hydrogen production are still not economically appealing. Then, new catalysts, for example for water oxidation, have been proposed to reduce the reactions’ activation energy [79,80]. Moreover, radically new catalysts, technologies, and approaches are still required [81]. Fuel cells, which can be considered electrochemical devices using fuel oxidation to convert chemical energy into electrical energy and simultaneously lower the amount of oxidant, are also widely studied [82].

After this brief excursus about sustainable materials developed by INSTM researchers, it is evident that some of the proposed materials may be attributed to multiple categories. For example, new synthesized materials (that were classified in the chemicals section) may have biological applications or act as a catalyst for energy production [83]. Indeed, the bio-refinery approach was introduced to solve the future energy and environmental needs, but renewable feedstocks are currently exploited both for fuels and chemicals production [84]. Then, it is clear that there is a significant interconnection among the materials categories as well as there is an interdependence among the three sustainability pillars and the development targets. In Table 2, by a way of example, some papers have been reported according to relevant SDGs’ Targets and the key contribution that sustainable materials can provide. 

## 4. An Analysis of the SDGs and Sustainable Materials Interconnections

The development of sustainable materials is strongly connected with the achievement of several SDGs. Power generation by renewable sources, new clean fuels, and the development of new batteries are all strictly dependent on the materials they are based on (SDG 7: Affordable and Clean Energy). Moreover, carbon capture and sequestration and the preservation of clean air and water need sustainable technologies, and this depends on the materials used in the processes (SDG 6: Clean Water and Sanitation and SDG 11: Sustainable Cities and Communities). The use of waste and by-products to produce materials in substitution to raw materials is the only way to preserve natural resources [49]. For example, the energy usage by the mining industries globally, to provide raw materials, is estimated to be around 11% of total energy consumption [85]. Then, the extraction industry is highly energy-intensive and needs to be better managed, not only by reforming its energy usage, but also by reducing the extraction of natural resources. Europe has the ambition to move towards cleaner energy delivery, with also the aim to decrease the pollution related to the energy sector. For this aim, decoupling energy consumption from carbon dioxide production is mandatory (SDG 13: Climate Action).

In this frame, it is fundamental to highlight that the management strategy involving sustainable materials differs from the circular economy concept and zero-waste approach, because it is based on the net sustainability advantages over the possible final material disposition and/or natural resources extraction [86]. For example, it is not always clear that it will be extremely hard to completely avoid any raw materials mining, due to the fundamental importance of some elements (like Cu, and rare earth elements) for renewable electricity generation technologies and/or for transmission and distribution infrastructures [18]. Indeed, although the Paris Agreement, signed in 2016 within the United Nations Framework Convention on Climate Change, suggested limiting the levels of average global temperature increase, an expansion of some mining sectors on an unprecedented scale is envisaged, to secure the necessary basic materials to build renewable energy infrastructure and electric transport [87]. Then, to guarantee the sustainability of the next future energy markets, the sustainable twenty-first-century economy is expected to be still highly dependent on mined elements [18]. Obviously, recycling of several resources will help to achieve the SDGs (SDG 12: Responsible Consumption and Production). Moreover, it is evident that materials management strategies must be addressed to encompass all the sustainability impacts, with the by-product that circular economy principles cannot be always guaranteed.

Table 2 shows that sustainable materials can be strictly interconnected with almost all the SDGs, as was shown by applicative examples in Section 3.

The SDGs’ interconnection implies that, in some cases, to promote suitable measures in a sector to support a specific goal, it is mandatory to also foresee interventions in other sectors. For example, raw materials, energy, and resources saving are the main determinants of technological progress, and they are also the main interests of any entrepreneur aiming for profit (SDG 9: Industry, Innovation, and Infrastructure). Nevertheless, often, a company′s economic approach is not based on sustainable growth principles. Then, suitable actions and/or approaches to promote a combination of economic growth and implementation of ecological responsibilities need to be introduced. In this frame, eco-design, which must take into account several complex technical objectives to manage raw materials, is not only connected with repair and industrial modernization (with also the derived social implications), but it is also connected with Earth resources conservation, recycling of materials, and waste, air, water, and soil pollution. The introduction of a new simple approach to evaluate the raw materials substitution sustainability [46,88,89] represents a fundamental step to accelerate the transition toward circular economy principles and support for small companies that often require a fast low-cost validation of a new technology developed at a laboratory scale.

Table 2 clearly shows the strict dependence of SDGs’ achievements from materials. Moreover, even for SDGs not directly related to materials (not reported in Table 2), indirect effects due to the diffusion of sustainable materials can be envisaged.

For example, the production of all the sustainable materials, based on the principles to minimize their impact on the environment, society, and the economy throughout their life cycle [90], is in accord with the SDG 14 (Life Below Water) and 15 (Life on Land) targets, devoted to preserving marine resources and protecting and restoring the ecosystems. 

Sustainable materials are also connected to SDG 4 (Quality Education). Indeed, research activities on these materials not only are based on continuous materials improvements, but also need the diffusion of the results, thanks to the involvement in dissemination projects and strategies (with the aim to diffuse the sustainability policies). Additionally, addressing sustainability in several application fields (such as materials for energy) allows the proposal of real examples of education facilities, enhances the quality of the instruction, promotes public awareness, and supports the sustainability concept diffusion. 

For example, access to scientific knowledge and derived information is mandatory to enable farmers to address the newly developed fertilizers, with the result to enhance the soil quality and the productivity of crops [18].

Sustainable materials apparently do not involve SDG 5 (Gender Equality). However, in mineral-rich countries, where raw materials are extracted, gender inequality is more evident than in other areas. For example, women cannot participate in mining activities concerning virgin materials extraction, and they are often the main victims of wars. Indeed, the mining sector is generally characterized by gender-based violence, especially in conflict-intense countries [18]. Additionally, due to longstanding experiences of oppression, it was shown that women more readily identify with the exploitation of natural resources by humans and have a stake in ending this exploitation [91]. On the contrary, some results of several research activities have clearly also shown that political conservatives (generally white males) have stronger inclinations to justify the existing system, in comparison to liberals [91], with the result of the denial or minimization of problems associated with sustainability. Increased participation of women in national policy contests has been associated with an increase in ratification of environmental treaties [91]. A recent study revealed that about 30% of the world population has no access to piped water [92]. For example, women and girls of sub-Saharan Africa spent most of their time collecting water at great distance from their household. The installation of piped water near the home promotes gender equality, providing the female gender with time to spend on their health and economic development. 

This strongly suggests that the role of women in science (for example in the development of new sustainable materials), where gender equality should be more easily guaranteed, is a key factor in determining support for sustainability policies. As a consequence, a continuous improvement of the sustainable materials sector can contribute to the rising of SDG 5. 

The sustainable materials issue also involves SDG 10 (Reduced Inequalities) and 16 (Peace, Justice, and Strong Institutions), since as already discussed, several world conflicts and inequalities involve the exploitation of natural resources [93]. In particular, resource extraction occurs where the concentration of minerals can be found, which often happens in developing countries or remote regions, with geopolitical instability. For example, between 1990 and 2009, at least 18 violent conflicts have been generated by the exploitation of natural resources, and over the last 60 years, at least 40% of all intrastate conflicts were linked to natural resources [93].

## 5. Future Perspectives

The COVID-19 crisis has shown that it is possible to reduce greenhouse gas (GHG) emissions [94] and to give more space to nature, but it is also shown that this happened in an unsustainable way: the sanitary crisis was coupled with a strong economic crisis, with high implications also for the social pillar of sustainability. The pandemic has demonstrated that the as-conceived SDGs are not resilient [95]. It is now necessary to recalibrate some of them. This is a very complicated issue, but it will be probably one of the main aims of the United Nations in the next months. In the development and recalibration of SDGs, it is evident that sustainable materials will be fundamental in all the possible scenarios devoted to sustainable development.

Moreover, considering the possible future activities in the development of new materials, there are several barriers to the adoption of sustainable materials. They include the incapability to account for the benefits of their use [96], the level of required knowledge and skill, cultural factors, import dependency, challenges of initial cost, and long payback period, the initial difficulties in the adoption of new methods and technology, and lack of suitable legislation [97] able to promote their use [98]. For example, manufacturing companies may have little knowledge about their responsibility for the impact connected with their designs and/or materials choices, and the advantages that new materials may offer them.

To face and reduce these barriers, it is fundamental to enhance the knowledge and the dissemination of the new opportunities to all the potential stakeholders.

In this context, SDG 17 (Partnership for the Goals) encourages action to work more holistically across sustainability goals. Then, due to the connections of all SDGs with materials and resources, it would be stimulating to propose a “Sustainable Materials Partnership” involving different stakeholders (from industries to people), in order to support the achievement of SDGs in all the countries. A primitive example concerning the possibility to conjugate materials with SDGs may be found in the International Council on Mining and Metals guidelines, which proposed mining companies implementing some social actions to improve the education and well-being of local populations resident in the territories where raw materials were extracted [18]. This was expressly connected to raw material mining, but other examples may link with other specific materials. For example, the introduction of a partnership for the development of high-quality sustainable fertilizers may generate a new baseline for the production of new sustainable materials able not only to promote food security (SDG 2: Zero Hunger), but also to act as a catalyst mechanism for agriculture development, with the increase of population well-being (SDG 3: Good Health and Well-Being). This may produce specific synergic effects on poverty alleviation (SDG 1: No Poverty), and socio-economic and social conditions linked to agriculture workers (in some countries) (SDG 8: Decent Work and Economic Growth). 

More generally, the partnership may be addressed to all sustainable materials. This will allow having a framework not only devoted to support specific SDGs, but also a challenging mechanism to promote the overall achievement of all SDGs. While a partnership may be an unsuitable tool to relieve the limited resources trouble, it may be a vehicle for more suitable resource management.

The new proposed partnerships should be multi-trans-disciplinary, reaching to promote SDGs at all levels. The first activity to address these challenges may be the establishment of an international network of experts or expert institutions on sustainable materials, as already recently proposed for better management of anthropogenic resources [99]. These experts should be aware of all the programs and projects, developed in their country, concerning materials, to better address the connection with the SDGs and monitor the progress of their achievement, due to the diffusion of the sustainable material. It would be proposed to create a multi-criteria tool based on sustainable materials use, able to estimate the advances in specific SDGs’ indicators, that may occur by using the proposed new materials, for example as a substitute for mined resources. 

Like the SDGs, a partnership would need the definition of suitable indicators and measurable goals. This tool should be able to take into account the possible contribution of a single material on all the SDGs’ indicators. Additionally, with the support of applied research in specific fields (i.e., considering real case studies), it would be possible to validate this tool and better understand the barriers and the limitations to achieve 2030 agenda aims. This partnership may find political authorities as the main interlocutor.

## 6. Conclusions

The 2030 agenda for sustainable development was designed to achieve the SDGs, balancing three dimensions of sustainable development: economic growth, environmental protection, and social inclusion. This work, based on an Italian experience, shows the opportunities that sustainable materials offer to achieve the SDGs’ fulfillment. The enhancement of the knowledge and the recognition of the SDGs’ interactions are essential to get the final goal of sustainability for the next generations of humans on this planet, allowing synergic paths among different research disciplines to contribute to different SDGs. This paper reviews INSTM researches about materials and sustainability, not only to highlight the materials’ importance to promote sustainable development, but also to stress that materials represent an optimum example about the SDGs’ interconnections. All the considered materials can be grouped in the following categories: chemicals, composites, novel materials for pollutants sequestration, bio-based and food-based materials, materials for green building, and materials for energy. However, the barriers to reach the SDGs are also presented. In this frame, it is suggested that the establishment of a partnership on sustainable materials may be a winning strategy to achieve the mutual interacting objectives of different SDGs. A partnership, made by an international multi-trans-disciplinary network of experts, is proposed as a vehicle for more suitable resource management. 

Finally, it is possible to present several other opportunities that sustainable materials can offer in the future. They can be resumed in:

Innovation and market development: increase the use of renewables, shifting business models away from carbon-intensive fuels. Develop new products with lower environmental and health impacts. Support circular economy transition.

Efficiency and cost savings: support materials efficiency strategies, by eco-design approaches.

Reputation management: avoid in some cases potential negative social and environmental impacts associated with the extraction of the raw material and allow better management of the supply risks of some limited resources.

Risk reduction: promote sustainable management to limit natural resources depletion.

Build a new world: in all the new research activities, it is imperative to pay great attention to sustainable urbanization strategies, biodiversity preservation, energy and water use, waste recycling, and economic and social development. 

The post-COVID-19 scenario will depend on the world capability in pursuing the positive changes established by SDGs and building on them [100]. These will be achieved if global efforts will be realized toward sustainable development.

## Figures and Tables

**Table 1:** Summary of papers (identified by DOI name) concerning sustainable materials developed by INSTM researchers grouped in 6 categories and their SDGs associated.

Categories	SDGs	DOI
Chemicals	8; 12; 13; 15	10.1002/chem.201802903
	7; 12; 13	10.1016/j.jcou.2019.10.009
	7; 9; 13	10.1007/s11244-018-1003-5
	7; 9; 12; 13	10.3390/catal8010041
	7; 9; 11; 13	10.1021/acscatal.0c01989
	2; 6; 14; 15	10.1007/s41348-016-0036-x
	7; 9; 11; 13	10.1021/acs.langmuir.8b00642
	11; 13	10.1021/acsmacrolett.0c00495
	11; 13	10.3390/molecules25163717
	1; 7; 8; 9; 11; 12	10.1002/ejoc.202000140
	9; 12	10.1039/c9gc01071h
	1; 2; 12; 13; 15	10.1021/acssuschemeng.8b00215
	9	10.1055/s-0036-1591937
	7; 9	10.1021/acsaem.9b00132
	7; 9; 13	10.1039/c6cy00942e
	7; 9	10.1016/j.cogsc.2019.07.008
	7; 9; 13	10.1002/cssc.201701506
	7; 9; 13	10.1016/j.jcou.2017.08.008
	7; 9; 13	10.1016/j.cattod.2015.07.020
	7; 9; 13	10.1073/pnas.1524806113
	2; 7; 12; 13; 15	10.1021/acscatal.6b00101
	2; 9; 12	10.1021/acssuschemeng.5b00806
	7; 9; 12; 13	10.1002/cssc.201600202
	2; 8; 15	10.1515/pac-2018-1223
	7; 9; 13	10.1039/c6cp08053g
	12; 13	10.1016/j.jcou.2017.01.030
	3; 9; 12	10.1002/cctc.201601547
	8; 11; 12; 15	10.1021/acssuschemeng.7b00410
	9; 12; 13	10.1002/celc.201700104
	3; 9	10.1021/acssuschemeng.7b00133
	9	10.1021/jacs.7b03412
	7; 9	10.1039/c7ta02316b
	7; 9; 12; 13	10.1016/j.apcata.2017.07.010
	11; 12; 13; 15	10.1039/c7gc02678a
	2; 3; 9; 12; 14; 15	10.1063/1.5047771
	7; 9; 12	10.1016/bs.acat.2018.10.001
	9	10.1039/c5ra23135c
	9	10.1039/c6ra18200c
	9; 11	10.1039/c6nj02199a
	8; 9; 15	10.1016/j.msea.2017.04.016
	7; 13	10.1021/acssuschemeng.7b01742
	3	10.1039/c8ra04315a
	3; 12; 13	10.3144/expresspolymlett.2018.14
	7; 12; 13	10.1016/j.jcou.2018.01.028
	2; 3; 12; 15	10.1021/acscatal.7b03843
	3; 9; 12	10.1016/j.jiec.2017.10.003
	7; 9; 11; 13	10.1016/j.cattod.2017.09.048
	7; 9; 12; 13	10.1016/j.cattod.2017.08.060
	9; 11	10.1039/c8gc00086g
	9; 12; 15	10.1021/acssuschemeng.8b00840
	9	10.1021/acs.jpcc.7b11473
	9	10.3390/ma11071051
	3; 6; 14	10.3390/nano8070488
	7; 9; 13	10.1016/j.apcatb.2018.01.049
	9	10.1166/jnn.2018.15385
	9	10.1039/c7fd00211d
	9	10.1039/c7cy02309j
	7; 9	10.1002/admi.201800070
	3; 7; 8; 9	10.1039/c8gc01248b
	9	10.1021/acs.inorgchem.8b01101
	7; 9; 11; 13	10.1055/s-0037-1610331
	9	10.1021/acssuschemeng.8b03101
	9; 11	10.1016/j.scitotenv.2018.07.188
	9; 11; 12	10.1016/j.jiec.2018.07.046
	8; 9; 10; 11; 12; 15; 16	10.1021/acssuschemeng.8b04983
	9	10.1016/j.jlumin.2018.10.106
	9; 12	10.1002/ejic.201801334
	9; 12	10.1002/admi.201801874
	3; 6; 8; 9; 10; 12; 14	10.1002/adma.201802920
	8; 9; 12	10.1002/cssc.201900238
	11; 12	10.1021/acsanm.9b00487
	9	10.1016/j.apcata.2019.05.015
	9	10.1021/acsami.9b04599
	9; 12	10.1021/acssuschemeng.9b02402
	11; 12; 15	10.1021/acssuschemeng.9b01877
	3; 8; 9	10.1515/psr-2018-0097
	8; 9; 12; 13	10.1002/cssc.201901352
	7; 9; 13	10.1002/adsu.201900046
	3; 6; 14	10.1016/j.colsurfa.2019.123634
	9	10.1063/1.5130400
	9	10.1021/acsaem.9b01773
	9	10.1039/c9nr05947d
	9	10.1039/c9ce01462d
	9	10.1002/sia.6601
	12; 13	10.1016/j.jcou.2019.09.017
	9; 11; 12; 13; 15	10.1149/1945-7111/abb0f2
	3; 12; 13	10.1016/j.jbiotec.2019.12.005
	9; 12	10.1016/j.msea.2019.138879
	7; 8; 9; 12; 13	10.3390/catal10020191
	9; 12	10.1039/c9se00847k
	9; 12	10.1021/acs.jpcc.9b11323
	9	10.3389/fchem.2020.00272
	2; 7; 9; 11; 12; 13; 14; 15	10.3390/polym12071558
	7; 12	10.1039/c7gc01749a
	7; 9; 11; 12; 15	10.1016/j.electacta.2017.07.005
	7; 9; 12; 13	10.1016/j.apcatb.2017.01.056
	2	10.3390/nano10061174
	2; 6; 9; 12; 14; 15	10.1016/j.eurpolymj.2019.109389
	8; 9; 11	10.3390/polym8090319
	2; 9; 11; 13; 14; 15	10.1016/j.polymdegradstab.2016.10.001
	9	10.1021/acs.oprd.0c00285
	9	10.1021/acs.jpcc.0c07498
	9	10.1016/j.optmat.2020.110234
	8; 12; 15	10.1021/acssuschemeng.0c03218
	2; 12; 11; 14; 15	10.1007/s10924-020-01843-3
	4; 11; 12; 15	10.3390/polym12061212
	4; 13; 14	10.3390/su12124939
	3	10.1021/acsami.0c05252
	9	10.1063/5.0007045
	9	10.1021/acssuschemeng.9b05325
	2; 3; 6; 9; 11; 12; 13; 15	10.1002/ldr.2915
	
Composites	9; 11; 12; 15	10.3390/polym12123054
	9; 11; 12; 15	10.1002/pen.25615
	9	10.1016/j.carbon.2020.05.084
	2; 8; 11; 12; 13; 15	10.3390/polym10111256
	1; 2; 9; 12; 13	10.1021/acssuschemeng.8b01202
	9; 12; 13; 15	10.2174/1385272822666180515120948
	2; 4; 9; 12; 13; 15	10.1016/j.eurpolymj.2016.04.013
	1; 2; 7; 8; 9; 11; 12; 15	10.3390/ijms20040960
	11; 12; 15	10.1016/j.compositesb.2017.08.012
	4; 11; 12; 15	10.1016/j.wasman.2019.11.031
	2; 9; 11; 12; 14; 15	10.5301/jabfm.5000335
	2; 4; 7; 8; 9; 11; 12; 13; 14; 15	10.3390/su12104193
	7; 9; 12	10.1504/IJSA.2017.086848
	3; 8; 11; 12; 13; 15	10.1016/j.scitotenv.2018.04.150
	2; 7; 9; 11; 12; 13; 14; 15	10.3390/ma13214892
	11; 12; 15	10.1016/j.wasman.2020.05.054
	11; 13; 15	10.1007/s10853-015-9710-9
	4; 8; 9; 11; 12; 15	10.1016/j.jclepro.2017.06.028
	4; 8; 9; 11; 12; 15	10.1088/1757-899X/329/1/012001
	3; 9; 11; 12; 15	10.1063/1.5045886
	9; 12; 13; 15	10.1016/j.mtcomm.2018.02.007
	9; 11; 12; 13	10.3390/nano9010046
	2; 11; 12; 15	10.3390/ijms20030504
	9; 11; 12; 15	10.1177/2280800019831224
	2; 12	10.1016/j.foodcont.2018.10.027
	9	10.1016/j.synthmet.2019.116201
	3; 9	10.1111/jiec.12968
	9; 11; 12; 13	10.1016/j.jcis.2019.10.083
	8; 9; 11; 12	10.3390/ma13051149
	11; 12; 15	10.3390/su12093561
	3; 6; 11; 12	10.1016/j.lwt.2020.110088
	9; 11; 12	10.3390/app10196674
	3; 6; 7; 11; 12; 13; 15	10.3390/app10030754
	9	10.1021/acsanm.9b00366
	8; 9; 11; 12; 15	10.1007/s10570-018-1745-z
	3; 6; 11; 12; 15	10.1016/j.envpol.2016.04.053
	7; 8; 9; 11; 13	10.1002/ente.201600609
	2; 9; 11; 12	10.1002/pi.5090
Novel materials for pollutants sequestration	3; 6; 11; 14	10.1016/j.apsusc.2020.147394
	3; 6; 14	10.1016/j.cattod.2019.06.034
	6; 9; 11; 14	10.1002/adsu.201900112
	3; 11; 12	10.1002/slct.201601719
	6; 7; 9; 11; 13; 14	10.1016/j.jpowsour.2017.02.084
	6; 11; 14	10.3390/ma12244091
	3; 6; 7; 11; 12; 13; 14; 15	10.3390/ma13183964
	6; 11; 12; 15	10.1016/j.arabjc.2014.04.006
	3; 6; 7; 11; 12; 13; 15	10.3389/fchem.2018.00534
	6; 7; 12; 13; 14; 15	10.1016/j.jclepro.2016.09.070
	3; 7; 12; 13; 15	10.4028/www.scientific.net/MSF.941.2237
	3; 6; 7; 12; 13; 14; 15	10.3389/fchem.2018.00060
	3; 7; 11; 12; 13; 15	10.1155/2019/1732196
	3; 6; 7; 11; 12; 14; 15	10.1016/j.jenvman.2019.05.104
	3; 6; 7; 11; 12; 13; 15	10.3390/app10176075
	2; 3; 6; 7; 11; 12; 13; 15	10.1016/j.wasman.2020.05.010
	3; 6; 11; 12; 14; 15	10.1007/s11157-016-9416-8
	6; 11; 12; 14; 15	10.1007/s11270-020-04671-2
	3; 9; 11; 12; 15	10.1021/acssuschemeng.6b01294
	3; 9; 11; 12; 13; 15	10.1007/s11356-017-9037-y
	3; 6; 9; 11; 14; 15	10.1016/j.ecoenv.2018.02.037
	2; 6; 8; 11; 12; 14; 15	10.3390/nano9020162
	2; 6; 8; 11; 12; 14	10.3389/fchem.2020.00763
	3; 9; 11	10.3390/ma12060937
	3; 6; 11; 14	10.1016/j.apcatb.2015.08.006
	3; 6; 7; 11; 12; 13; 15	10.3390/ma12172714
Bio-based and food-based materials	3; 9	10.1016/j.jbiotec.2020.11.001
	3	10.1007/s10971-017-4446-4
	3; 11; 12; 13; 14; 15	10.3390/jfb11020023
	3; 11; 12; 14; 15	10.3390/polym12061366
	3; 11; 12	10.1021/acssuschemeng.6b02850
	8: 9; 11	10.3390/molecules25184046
	2; 7; 9; 11; 12; 13; 14; 15	10.1016/j.jclepro.2018.07.252
	2; 3; 6; 8; 11; 12; 13; 14	10.3390/su10103547
	2; 7; 9; 11; 12; 13; 14; 15	10.3390/polym12081641
	2; 8; 11; 12; 15	10.3390/s19040801
	7; 9; 11; 12; 13	10.3303/CET1650044
	2; 3; 9; 11; 12; 13; 15	10.1063/1.4949586
	2; 3; 11; 12; 15	10.1002/macp.201500353
	3; 11; 15	10.1002/marc.201900660
	2; 3; 11	10.1038/NNANO.2017.58
	8; 9; 12	10.1016/j.apcatb.2017.04.007
	3	10.1021/acsami.7b11839
	2; 12; 13; 15	10.1021/acssuschemeng.7b03782
	9; 12	10.1021/acssuschemeng.8b00600
	1; 2; 9; 11; 12; 14; 15	10.3390/ma12091476
	3	10.1177/0883911519843309
	8; 9; 11; 12; 15	10.1016/j.jcis.2019.03.055
	9; 12	10.1021/acssuschemeng.9b02177
	2; 8; 9; 11; 12; 13; 15	10.3390/polym11122118
	3	10.1016/j.msec.2019.110166
	3	10.1039/c9bm01007f
	3; 4; 9	10.1016/j.carbpol.2020.116502
	2; 9; 11; 12; 14; 15	10.1021/acssuschemeng.0c03365
	3; 4	10.1039/d0py00843e
	2; 12; 15	10.3390/molecules25143313
	3; 9	10.1021/acs.analchem.0c00651
	2; 3; 9; 12	10.1177/1934578X160110032
Materials for green building	11; 12; 13; 15	10.3390/ma13132919
	11; 12; 13; 15	10.1016/j.jclepro.2019.119588
	15	10.1016/j.cemconcomp.2017.11.016
	9; 11; 12; 13; 15	10.1016/j.compositesb.2017.02.004
	7; 11; 12; 13; 15	10.3390/ma9060461
	9; 11; 12; 13; 15	10.1016/j.conbuildmat.2020.118682
	7; 11; 12; 13; 15	10.1016/j.jclepro.2019.02.160
	7; 9; 11; 12; 13; 15	10.1016/j.conbuildmat.2018.07.221
	4; 11; 12; 13; 15	10.1088/1757-899X/442/1/012024
	11; 12; 13; 15	10.1016/j.conbuildmat.2020.118436
	3; 7; 11; 13; 15	10.1080/21650373.2019.1615012
	3; 11	10.3390/molecules24234226
	11; 12; 13; 15	10.1155/2018/8676708
	9; 11; 12; 15	10.1155/2018/5256741
	9; 11; 12; 13; 15	10.1016/j.conbuildmat.2016.12.039
	3; 9; 11; 12; 13; 15	10.1016/j.scs.2019.101961
	3; 9; 11; 12; 13; 15	10.3390/app10228086
	11; 12; 13; 15	10.3390/su12155993
	3; 7; 11; 12; 13; 15	10.1016/j.jenvman.2018.04.081
	7; 8; 9; 11; 13; 15	10.1016/j.conbuildmat.2018.04.034
	9; 11; 12; 15	10.1016/j.ceramint.2015.12.002
	11; 12; 13; 15	10.3390/su10030874
	11; 12	10.1016/j.conbuildmat.2018.03.137
	8; 9; 11; 12; 15	10.3390/su10114013
	8; 11; 12; 13; 15	10.1016/j.jclepro.2019.04.299
	8; 9; 11; 13; 12; 15	10.3390/su12239916
	7; 8; 9; 11; 13	10.1063/5.0012139
	9; 11	10.3390/buildings10060105
Materials for energy	7; 9; 11; 12; 13	10.1002/ejoc.202001296
	7; 9; 11; 13	10.1016/j.ijhydene.2020.07.049
	7; 9; 11; 13	10.1021/acs.nanolett.0c00594
	7; 9	10.1021/acsaem.9b00657
	3; 4; 7; 8; 11; 12; 13	10.1002/cctc.201700489
	7; 9; 13	10.1038/ncomms13549
	7; 9	10.1002/cssc.201802637
	7; 9; 11; 12; 13	10.1016/j.cattod.2018.03.005
	7; 9; 11; 13	10.1002/adma.201801712
	7; 9; 13	10.1016/j.apcatb.2017.09.071
	7; 9; 11; 13	10.1016/j.cattod.2017.08.036
	7; 9; 13	10.1016/j.jechem.2016.11.004
	7; 8; 9; 13	10.1002/cssc.201501059
	7;9	10.1063/1.5134466
	7; 9; 12; 13	10.1016/j.nanoen.2018.05.070
	7; 8; 12; 13	10.1016/j.ijhydene.2019.07.201
	7; 8; 9; 11; 12; 13; 15	10.1039/c9sc05596g
	7; 9; 11; 13	10.1021/acssuschemeng.0c02373
	7; 9; 11; 13	10.1002/cctc.202000999
	7; 9; 13	10.1039/c7se00005g
	9	10.1021/acsaem.9b0177
	7; 9; 13	10.1039/c5gc02139a
	7; 9; 11; 13	10.3390/nano10081585
	7; 9; 11; 12; 13	10.1039/c9dt00790c
	7; 9; 11; 12; 13	10.1039/c9dt01448a
	7; 9; 13	10.1016/j.solmat.2018.01.007
	7; 9; 12; 13	10.1021/acsaem.7b00196
	7; 9; 12; 13	10.3390/en10091394
	7; 9; 11; 13	10.1557/mre.2020.37
	7; 8; 11; 12; 13	10.3390/en13174299
	7; 9; 11; 12; 13; 15	10.1016/j.seta.2018.03.006
	3; 6; 7; 9; 11; 12; 13; 14; 15	10.3390/molecules25235620
	7; 13	10.3390/su10114225
	7; 9; 11; 13	10.1016/j.mssp.2015.07.051
	7; 9; 11; 12; 13	10.1021/acs.iecr.6b00134
	2; 7; 9; 11; 12; 13	10.1016/j.apcata.2015.09.022
	7; 9; 11; 13	10.1021/acsanm.0c01951
	7; 9; 11; 13; 15	10.3390/ma10030325
	7; 8; 11; 12; 13; 15	10.1016/j.renene.2016.07.040
	7; 11; 12; 13; 15	10.1016/j.ijhydene.2016.07.149
	7; 8; 9; 11; 12	10.1039/c6gc02625g
	7; 8; 9; 12; 13	10.1002/ejoc.201600653
	7; 9; 11; 13	10.1117/12.2273369
	7; 9; 11; 13	10.1002/ente.201600420
	7; 8; 9; 12; 13	10.1016/j.jechem.2017.03.004
	7; 8; 9; 13	10.1002/cctc.201601659
	7; 9; 13	10.1039/c7se00075h
	7; 9; 13	10.1016/j.cej.2017.03.066
	7; 8; 9; 13	10.1016/j.solmat.2017.04.011
	7; 8; 9; 13	10.1021/acs.energyfuels.7b02434
	7; 9; 11; 13	10.1021/acs.inorgchem.7b02323
	7; 8; 9; 12; 13	10.1039/c7cy02099f
	7; 9; 13	10.1039/c8ra08880b
	7; 9; 13	10.1002/cssc.201701707
	7; 9; 11; 13	10.1016/j.solener.2018.02.009
	7; 9; 11; 13	10.1115/1.4038415
	7; 9; 11; 12; 13	10.1021/acssuschemeng.8b00144
	7; 9; 12; 13	10.1016/j.fuel.2018.03.137
	7; 8; 9; 13	10.1016/j.jpowsour.2018.07.002
	7; 9; 12; 13	10.1016/j.electacta.2018.07.148
	7; 9; 11; 13	10.1021/acssuschemeng.8b02103
	7; 9; 11; 13	10.1016/j.electacta.2018.09.204
	7; 9; 11; 12; 13	10.1002/chem.201803668
	7; 8; 9; 11; 12; 13	10.1021/acsaem.8b01361
	14	10.1038/s41598-019-45926-1
	7; 9; 11; 12; 15	10.1016/j.jallcom.2018.10.201
	7; 9; 11	10.1016/j.electacta.2019.03.167
	7; 8; 9; 11; 12; 13	10.1007/s11581-019-02878-w
	7; 9; 12; 13	10.1039/c9ra03435h
	4; 7; 13	10.1080/17477778.2019.1679612
	7; 9; 12	10.1149/1945-7111/ab6c59
	7; 9; 11; 12; 13	10.3390/polym12030720
	7; 9	10.1002/anie.201913578
	2; 7; 12; 13; 15	10.1016/j.cattod.2019.12.009
	7; 8; 9; 11; 12; 13; 15	10.3390/molecules25092200
	7; 8; 9; 11; 12; 13	10.1149/1945-7111/ab856e
	7; 8; 9; 11; 13	10.1039/d0gc01148g
	7; 9; 11; 13	10.1016/j.scib.2020.06.015
	7; 9; 11; 13	10.1021/acssuschemeng.0c05235
	7; 8; 9; 11	10.1016/j.rser.2020.110105
	7; 9; 11; 13	10.1002/adsu.202000177
	7; 9; 11; 12; 13	10.1002/cssc.202001885
	7; 8; 9; 11; 12; 13	10.1007/s10668-020-00891-y
	11; 12; 13; 15	10.1016/j.jeurceramsoc.2019.11.068
	7; 9; 11; 13	10.1021/acssuschemeng.0c02623
	7; 8; 9; 11; 12; 13	10.3390/nano10081588
	7; 9; 11; 12; 13	10.1016/j.jcis.2020.03.092
	7; 9; 11	10.1039/d0se00134a
	7; 9; 11; 13	10.1016/j.ijhydene.2018.01.201
	9; 11; 12; 15	10.3390/ma13102284
	2; 7; 9; 12; 13	10.1007/s11244-018-1002-6
	7; 9; 11; 13	10.1021/acs.iecr.6b01581
	7; 9; 11; 13	10.1021/acsami.6b03345
	7; 9; 11; 13	10.1039/d0ta05972b
	7; 9; 13	10.1007/s11244-016-0547-5
	7; 9; 13	10.1016/j.ijhydene.2016.09.127
	7; 9; 13	10.1039/c7cy01067b
	2; 3; 6; 7; 11; 12; 13; 14	10.1016/j.heliyon.2018.e00560

**Table 2:** Selected papers (identified by DOI name) according to the relevant Sustainable Development Targets (of SDGs) with the key contribution that sustainable materials can provide.

GOAL 1: END POVERTY IN ALL ITS FORMS EVERYWHERE		
Relevant Sustainable Development Targets	Key Contributions	References (DOI)
1.4	Support the utilization of local materials (for example derived from waste), reducing the use of natural resources.	10.1002/ejoc.202000140
1.a	Promote the local green markets development.	10.3390/ijms20040960
GOAL 2: ZERO HUNGER		
Relevant Sustainable Development Targets	Key Contributions	References (DOI)
2.3	Develop new sustainable fertilizers, derived from organic waste, reducing the use of natural resources.	10.1002/ldr.2915
2.4	Reduce the use of pesticide, promoting sustainable agricultural materials’ use.	10.3390/nano10061174
2.4	Recover ammonia to produce chemicals.	10.1021/acssuschemeng.8b00215
GOAL 3: ENSURE HEALTHY LIVES AND PROMOTE WELL-BEING FOR ALL AT ALL AGES		
Relevant Sustainable Development Targets	Key Contributions	References (DOI)
3.1	Produce more sustainable and low-cost pharmaceutical materials, available for all the population.	10.1007/s10971-017-4446-4
3.9	Introduce sustainable materials, able to trap air particulate matter (PM), provide a better indoor environment, and minimize the health risks due to the air PM.	10.1016/j.scs.2019.101961
GOAL 4: ENSURE INCLUSIVE AND EQUITABLE QUALITY EDUCATION AND PROMOTE LIFELONG LEARNING OPPORTUNITIES FOR ALL		
Relevant Sustainable Development Targets	Key Contributions	References (DOI)
4.3	Support dissemination activities about the importance of using sustainable materials to support circular economy through education systems for the next generations.	10.3390/su12104193
GOAL 6: ENSURE THE AVAILABILITY AND SUSTAINABLE MANAGEMENT OF WATER AND SANITATION FOR ALL		
Relevant Sustainable Development Targets	Key Contributions	References (DOI)
6.3	Develop new sustainable products to improve water quality.	10.1002/adsu.201900112
6.4	Promote the use of sustainable materials, with the aim to reduce environmental pollution and minimize the release of hazardous chemicals.	10.3390/ma12244091
6.5	Support the use of sustainable phosphorous alternative to reduce water eutrophication.	10.1002/ldr.2915
GOAL 7: ENSURE ACCESS TO AFFORDABLE, RELIABLE, SUSTAINABLE, AND MODERN ENERGY FOR ALL		
Relevant Sustainable Development Targets	Key Contributions	References (DOI)
7.1	Reduce the energy need to extract and work natural resources.	10.1016/j.jenvman.2018.04.081
7.2	Identify suitable fuels able to avoid fossil sources.	10.3390/ma10030325
7.3	Reduce the greenhouse gas emissions need to extract and work natural resources.	10.1016/j.jclepro.2018.07.252
GOAL 8: PROMOTE SUSTAINED, INCLUSIVE, AND SUSTAINABLE ECONOMIC GROWTH, FULL AND PRODUCTIVE EMPLOYMENT, AND DECENT WORK FOR ALL		
Relevant Sustainable Development Targets	Key Contributions	References (DOI)
8.1	Propose the development of new green markets, based on the circular economy principles.	10.3390/su12104193
8.2	Introduce new tools able to evaluate the sustainability of new proposed technologies to speed their transfer.	10.1016/j.jclepro.2017.06.028
8.4	Promote the innovation in sustainable materials to lead to higher industrial productivity and more efficient use of natural resources.	10.3390/ijms20040960
8.6	Support the production of new second-hand materials, to increase the local resources productivity.	10.3390/en10091394
GOAL 9: BUILD RESILIENT INFRASTRUCTURE, PROMOTE INCLUSIVE AND SUSTAINABLE INDUSTRIALIZATION, AND FOSTER INNOVATION		
Relevant Sustainable Development Targets	Key Contributions	References (DOI)
9.2	Support the innovation in sustainable materials by patent application.	10.1016/j.jclepro.2019.118779
9.5	Promote the enhancement of scientific research for the upgrading of the technological capabilities of industrial sectors.	10.1039/c7ta02316b
GOAL 11: MAKE CITIES AND HUMAN SETTLEMENTS INCLUSIVE, SAFE, RESILIENT, AND SUSTAINABLE AGES		
Relevant Sustainable Development Targets	Key Contributions	References (DOI)
11.c	Allow the use of locally available resources and alternative materials to reduce both the cost and the impact of new goods on the environment.	10.3390/su12104193
11.6	Promote the development of new sustainable materials that can contribute to reduce the air particulate matter.	10.3389/fchem.2018.00534
11.6	Propose new technologies able to recycle waste and promote their reuse, as for example as a filler in composites production.	10.3390/polym10111256
11.6	Reduce the energy and investments needed for wastewater treatment technologies.	10.1016/j.jpowsour.2017.02.084
GOAL 12: ENSURE SUSTAINABLE CONSUMPTION AND PRODUCTION PATTERNS		
Relevant Sustainable Development Targets	Key Contributions	References (DOI)
12.1	Introduce new sustainable materials allow to reduce the use of chemical in their synthesis.	10.1021/acssuschemeng.6b02850
12.2	Introduce new sustainable materials that can be produced from local supplies, to achieve efficient utilization of available resources.	10.1002/fsn3.946
12.3	Introduce new materials derived from food waste.	10.3390/su10103547
12.4	Promote the use of second-hand materials that can be ecological, non-hazardous, non-polluting, and non-toxic materials. The use of these materials will allow the recycling of some wastes, minimizing their negative effect on the environment.	10.3390/molecules25184046
12.5	Promote the sustainable materials use, to obtain a major impact in reducing waste through recycling and reuse.	10.1016/bs.acat.2018.10.001
12.6	Promote the diffusion of sustainable materials, to encourage companies to implement sustainability in their projects and promote the development of new green market.	10.1002/ejoc.202000140
GOAL 13: TAKE URGENT ACTION TO COMBAT CLIMATE CHANGE AND ITS IMPACTS		
Relevant Sustainable Development Targets	Key Contributions	References (DOI)
13.1	Introduce new sustainable chemicals.	10.3390/ma12172714
13.3	Increase the knowledge about the available renewable energy sources.	10.1038/ncomms13549
GOAL 14: CONSERVE AND SUSTAINABLY USE THE OCEANS, SEAS, AND MARINE RESOURCES FOR SUSTAINABLE DEVELOPMENT		
Relevant Sustainable Development Targets	Key Contributions	References (DOI)
14.1	Promote the use of alternative materials that do not contain dangerous substances, to reduce the extraction of mined resources and help to achieve the sustainability of marine ecosystems.	10.3390/su12124939
GOAL 15: PROTECT, RESTORE, AND PROMOTE SUSTAINABLE USE OF TERRESTRIAL ECOSYSTEMS, SUSTAINABLY MANAGE FORESTS, COMBAT DESERTIFICATION, AND HALT AND REVERSE LAND DEGRADATION AND HALT BIODIVERSITY LOSS		
Relevant Sustainable Development Targets	Key Contributions	References (DOI)
15.1	Promote responsible alternative and sustainable materials to achieve sustainability and to ensure better conservation for natural resources.	10.1016/j.conbuildmat.2018.04.034

## Supplementary Materials

The following are available online, Table S1: List of all the papers published by the research INSTM units working in sustainable materials (Authors, articles title, source title, abstract, ORCIDs, publication year and DOI are reported).
